# Correction: Perceived barriers to physical activity and their predictors among adults in the Central Region in Saudi Arabia: Gender differences and cultural aspects

**DOI:** 10.1371/journal.pone.0331572

**Published:** 2025-09-03

**Authors:** Osama Abdelhay, Mohammad Altamimi, Qusai Abdelhay, Marwan Manajrah, Ayla M. Tourkmani, Mutaz Altamimi, Taghreed Altamimi

There is an error in affiliation 6 for author Taghreed Altamimi. The correct affiliation 6 is: Department of Software Engineering, Alfaisal University, Riyadh, Saudi Arabia.
