# Supplementary material for: Perceived barriers to physical activity and their predictors among adults in the Central Region in Saudi Arabia: Gender differences and cultural aspects
Source: PLoS One. 2025 Feb 7;20(2):e0318798. doi: 10.1371/journal.pone.0318798 (PMC11805373; doi:10.1371/journal.pone.0318798)
Supplement: S1 File — (DOCX) [file pone.0318798.s001.docx]

**Validity and Reliability Test for PBAQ**

The internal consistency of the questionnaire was assessed using Cronbach's alpha. The overall Cronbach's alpha for the PBAQ was 0.83, indicating good internal consistency. The Cronbach's alpha values for the individual sections and dimensions are as follows:

**Internal Barriers (18 items):** Cronbach's alpha = 0.81

- **Lack of Energy (3 items):** Cronbach's alpha = 0.79
- **Lack of Motivation (2 items):** Cronbach's alpha = 0.76
- **Lack of Self-Efficacy (8 items):** Cronbach's alpha = 0.83
- **Beliefs and Thoughts (3 items):** Cronbach's alpha = 0.78

**External Barriers (9 items):** Cronbach's alpha = 0.79

- **Lack of Facilities (3 items):** Cronbach's alpha = 0.75
- **Lack of Time (4 items):** Cronbach's alpha = 0.80
- **Other Barriers (2 items):** Cronbach's alpha = 0.72

As previously described, the Perceived Barriers to Being Active Questionnaire (PBAQ) comprises two main sections:

1. **Internal Barriers (18 items)** divided into four dimensions:
   - **Lack of Energy** (3 items)
   - **Lack of Motivation** (2 items)
   - **Lack of Self-Efficacy** (8 items)
   - **Beliefs and Thoughts** (3 items)
2. **External Barriers (9 items)** divided into three dimensions:
   - **Lack of Facilities** (3 items)
   - **Lack of Time** (4 items)
   - **Other Barriers** (2 items)

**Inter-Class Correlation Analysis:**

**Results:**

- **Correlations Among Internal Barrier Dimensions:**
  - **Lack of Energy & Lack of Motivation:** *r* = 0.58
  - **Lack of Energy & Lack of Self-Efficacy:** *r* = 0.47
  - **Lack of Energy & Beliefs and Thoughts:** *r* = 0.35
  - **Lack of Motivation & Lack of Self-Efficacy:** *r* = 0.62
  - **Lack of Motivation & Beliefs and Thoughts:** *r* = 0.30
  - **Lack of Self-Efficacy & Beliefs and Thoughts:** *r* = 0.38
- **Correlations Among External Barrier Dimensions:**
  - **Lack of Facilities & Lack of Time:** *r* = 0.33
  - **Lack of Facilities & Other Barriers:** *r* = 0.29
  - **Lack of Time & Other Barriers:** *r* = 0.40
- **Correlations Between Internal and External Barrier Dimensions:**
  - **Lack of Energy & Lack of Facilities:** *r* = 0.25
  - **Lack of Energy & Lack of Time:** *r* = 0.28
  - **Lack of Motivation & Lack of Facilities:** *r* = 0.22
  - **Lack of Motivation & Lack of Time:** *r* = 0.35
  - **Lack of Self-Efficacy & Lack of Facilities:** *r* = 0.28
  - **Beliefs and Thoughts & Lack of Facilities:** *r* = 0.20

**Interpretation:**

- **Within Internal Barriers:**
  - The moderate correlations (*r* = 0.30 to 0.62) among internal barrier dimensions suggest that while these constructs are related (e.g., lack of motivation may be associated with lack of self-efficacy), they are distinct enough to be considered separate dimensions.
- **Within External Barriers:**
  - The moderate correlations (*r* = 0.29 to 0.40) among external barrier dimensions indicate a reasonable degree of association, which is expected as they all pertain to external factors influencing physical activity.
- **Between Internal and External Barriers:**
  - The lower correlations (*r* = 0.20 to 0.35) between internal and external barrier dimensions suggest that these constructs are related but measure different aspects of perceived barriers. This distinction supports the theoretical framework that internal and external barriers are separate constructs impacting physical activity behaviour.
