# Supplementary material for: Perceived barriers to physical activity and their predictors among adults in the Central Region in Saudi Arabia: Gender differences and cultural aspects
Source: PLoS One. 2025 Feb 7;20(2):e0318798. doi: 10.1371/journal.pone.0318798 (PMC11805373; doi:10.1371/journal.pone.0318798)
Supplement: Attachment — Submitted filename: PONE-D-24-42926 - rev..pdf [file pone.0318798.s002.pdf]

PLOS ONE

Perceived Barriers to Physical Activity and Their Predictors among Saudi Adults in the Central Region: Gender Differences and Cultural Aspects.

--Manuscript Draft--

**Manuscript Number:**	PONE-D-24-42926
**Article Type:**	Research Article
**Full Title:**	Perceived Barriers to Physical Activity and Their Predictors among Saudi Adults in the Central Region: Gender Differences and Cultural Aspects.
**Short Title:**	Perceived Barriers to Physical Activity among Saudi Adults
**Corresponding Author:**	Osama Abdelhay, Ph.D.<br>Princess Sumaya University for Technology<br>Amman, Amman JORDAN
**Keywords:**	Barriers; physical activity; Gender Differences; Cultural Aspects; Saudi Arabia; community health
**Abstract:**	Objective<br><br>To assess the perceived barriers hindering physical activity among adult residents of Riyadh, Saudi Arabia, and to identify associated sociodemographic and health-related factors, focusing on gender differences and cultural aspects.<br><br>Methods<br><br>A cross-sectional survey was conducted from January 2022 to February 2023, involving 7,903 physically inactive participants aged 18 to 80. Participants were recruited using a two-stage cluster sampling method from the Central Region of Saudi Arabia. In the first stage, subregions based on the administrative distribution by the Medical Service Department were selected. In the second stage, private and public entities within these subregions were identified from governmental agency lists. Participants were then conveniently approached within these entities. Data were collected using a validated questionnaire assessing sociodemographic characteristics, health history, dietary habits, and perceived internal and external barriers to physical activity.<br><br>Results<br><br>Of the participants, 67.2% were male, with a mean age of 36.45±13.69 years. Approximately one-third (35%) reported experiencing at least one internal barrier to physical activity, while 64.3% reported 1–2 internal barriers. For external barriers, 76.5% faced 1–2 barriers. The most common internal barriers were laziness (40.2%) and lack of self-motivation (27.5%); the most prevalent external barriers were lack of facilities (20.2%) and long working hours (19.6%). Females were significantly more likely than males to report cultural reasons (odds ratio [OR]=4.83; 95% confidence interval [CI]: 4.06–5.76; p<0.001) and religious reasons (OR=3.31; 95% CI: 2.59–4.23; p<0.001) as internal barriers. Multivariate analysis revealed that females were 14% more likely than males to report external barriers to physical activity (OR=1.14; 95% CI: 1.04–1.25; p=0.018), suggesting gender plays a role in perceived external obstacles. Additionally, older age, higher body mass index, higher education level, being married, certain employment statuses, and the presence of chronic diseases were significantly associated with increased reporting of both internal and external barriers. These findings highlight the complex interplay of demographic and health-related factors influencing physical activity participation.<br><br>Conclusions<br><br>There is a high prevalence of both internal and external barriers to physical activity among Saudi adults, with notable gender differences influenced by cultural factors. Females were more likely to report cultural and religious reasons as barriers. Tailored policies and interventions are urgently needed to address these barriers, such as

	promoting gender-specific physical activity programs, integrating physical activity into workplaces, enhancing public facilities, and conducting culturally sensitive educational campaigns. Addressing both internal motivations and external obstacles is essential to increase physical activity levels and combat the rising burden of non-communicable diseases in Saudi Arabia.
Order of Authors:	Osama Abdelhay, Ph.D.
	Mohammad Altamimi, PhD
	Qusai Abdelhay, MD
	Marwan Manajrah, BSc
	Ayla Tourkmani Tourkmani, PhD
	Mutaz Altamimi, MSc
	Taghreed Altamimi, PhD
Additional Information:	
Question	Response
**Financial Disclosure**<br><br>Enter a financial disclosure statement that describes the sources of funding for the work included in this submission. Review the submission guidelines for detailed requirements. View published research articles from *PLOS ONE* for specific examples.<br><br>This statement is required for submission and **will appear in the published article** if the submission is accepted. Please make sure it is accurate.<br><br>**Funded studies**<br>Enter a statement with the following details:<br>• Initials of the authors who received each award<br>• Grant numbers awarded to each author<br>• The full name of each funder<br>• URL of each funder website<br>• Did the sponsors or funders play any role in the study design, data collection and analysis, decision to publish, or preparation of the manuscript?<br><br>Did you receive funding for this work?	The author(s) received no specific funding for this work.
**Competing Interests**<br><br>Use the instructions below to enter a competing interest statement for this	The authors have declared that no competing interests exist.

submission. On behalf of all authors, disclose any competing interests that could be perceived to bias this work—acknowledging all financial support and any other relevant financial or non-financial competing interests.<br><br>This statement is required for submission and **will appear in the published article** if the submission is accepted. Please make sure it is accurate and that any funding sources listed in your Funding Information later in the submission form are also declared in your Financial Disclosure statement.<br><br>View published research articles from *PLOS ONE* for specific examples.<br><br>**NO authors have competing interests**<br><br>Enter: *The authors have declared that no competing interests exist.*<br><br>**Authors with competing interests**<br><br>Enter competing interest details beginning with this statement:<br><br>*I have read the journal's policy and the authors of this manuscript have the following competing interests: [insert competing interests here]*<br><br>* typeset	
**Ethics Statement**<br><br>Enter an ethics statement for this submission. This statement is required if the study involved:<br><br>• Human participants<br>• Human specimens or tissue<br>• Vertebrate animals or cephalopods<br>• Vertebrate embryos or tissues<br>• Field research<br><br>Write "N/A" if the submission does not	This study was approved by Prince Sultan Military Medical City under IRB 106-10-20. Each participant was provided with written consent on the first based of the self-administrated questionnaire.

require an ethics statement.<br><br>General guidance is provided below. Consult the submission guidelines for detailed instructions. **Make sure that all information entered here is included in the Methods section of the manuscript.**<br><br>**Format for specific study types**<br><br>**Human Subject Research (involving human participants and/or tissue)**<br>• Give the name of the institutional review board or ethics committee that approved the study<br>• Include the approval number and/or a statement indicating approval of this research<br>• Indicate the form of consent obtained (written/oral) or the reason that consent was not obtained (e.g. the data were analyzed anonymously)<br><br>**Animal Research (involving vertebrate animals, embryos or tissues)**<br>• Provide the name of the Institutional Animal Care and Use Committee (IACUC) or other relevant ethics board that reviewed the study protocol, and indicate whether they approved this research or granted a formal waiver of ethical approval<br>• Include an approval number if one was obtained<br>• If the study involved *non-human primates*, add *additional details* about animal welfare and steps taken to ameliorate suffering<br>• If anesthesia, euthanasia, or any kind of animal sacrifice is part of the study, include briefly which substances and/or methods were applied<br><br>**Field Research**<br><br>Include the following details if this study involves the collection of plant, animal, or other materials from a natural setting:<br>• Field permit number<br>• Name of the institution or relevant body that granted permission	
**Data Availability**	No - some restrictions will apply

Powered by Editorial Manager® and ProduXion Manager® from Aries Systems Corporation

Authors are required to make all data underlying the findings described fully available, without restriction, and from the time of publication. PLOS allows rare exceptions to address legal and ethical concerns. See the PLOS Data Policy and FAQ for detailed information.<br><br>A Data Availability Statement describing where the data can be found is required at submission. Your answers to this question constitute the Data Availability Statement and **will be published in the article**, if accepted.<br><br>**Important:** Stating 'data available on request from the author' is not sufficient. If your data are only available upon request, select 'No' for the first question and explain your exceptional situation in the text box.<br><br>Do the authors confirm that all data underlying the findings described in their manuscript are fully available without restriction?	
**Describe where the data may be found in full sentences. If you are copying our sample text, replace any instances of XXX with the appropriate details.**<br><br>• If the data are **held or will be held in a public repository**, include URLs, accession numbers or DOIs. If this information will only be available after acceptance, indicate this by ticking the box below. For example: *All XXX files are available from the XXX database (accession number(s) XXX, XXX.)*.<br>• If the data are all contained **within the manuscript and/or Supporting Information files**, enter the following: *All relevant data are within the manuscript and its Supporting Information files.*<br>• If neither of these applies but you are able to provide **details of access elsewhere**, with or without limitations, please do so. For example:	The data used in this study is anonymized and owned by the Department of Family and Community Medicine. However, due to the presence of several demographic identifiers, the data is restricted from being shared publicly. Should additional data be required for review purposes, we are happy to discuss options for secure access in accordance with our institution's policies.

Data cannot be shared publicly because of [XXX]. Data are available from the XXX Institutional Data Access / Ethics Committee (contact via XXX) for researchers who meet the criteria for access to confidential data.<br><br>*The data underlying the results presented in the study are available from (include the name of the third party and contact information or URL).*<br>• This text is appropriate if the data are owned by a third party and authors do not have permission to share the data.<br><br>* typeset	
Additional data availability information:	

Powered by Editorial Manager® and ProduXion Manager® from Aries Systems Corporation

Manuscript Click here to access/download;Manuscript;Manuscript Final New.docx

Perceived Barriers to Physical Activity and Their Predictors among Saudi Adults in the Central Region: Gender Differences and Cultural Aspects.

Authors

1. Osama Abdelhay, PhD., Department of Data Science, King Hussein School of Computing Sciences, Princess Sumaya University for Technology, Amman, Jordan. Senior Biostatistician, Department of Family & Community Medicine, Prince Sultan Military Medical City, Riyadh, Saudi Arabia o.abdelhay@psut.edu.jo
2. Mohammad Altamimi, PhD., Department of Nutrition and Food Technology, An-Najah National University, Nablus, Palestine. m.altamimi@najah.edu
3. Qusai Abdelhay, M.D., Consultant Orthopaedic Surgeon, Department of Orthopaedic Surgery, Al-Bashir Hospital, Amman, Jordan. quqtamimi@gmail.com
4. Marwan Manajrah, B.Sc., Senior Registered Nurse, Department of Family & Community Medicine, Prince Sultan Military Medical City, Riyadh, Saudi Arabia marwan_manajreh@yahoo.com
5. Ayla Tourkmani, PharmaD, PhD., Doctor of Pharmacy and Clinical Pharmacist, Department of Family & Community Medicine, Prince Sultan Military Medical City, Riyadh, Saudi Arabia aturkmany@psmmc.med.sa
6. Mutaz Altamimi, MSc., Senior Registered Nurse, King Hussein Cancer Centre, Amman, Jordan. mutaztamimi@yahoo.com
7. Taghreed Altamimi, PhD., Department of Software Engineering, Al Faisal University, Riyadh, Saudi Arabia, taaltamimi@alfaisal.edu

Abstract

Objective: To assess the perceived barriers hindering physical activity among adult residents of Riyadh, Saudi Arabia, and to identify associated sociodemographic and health-related factors, focusing on gender differences and cultural aspects.

Methods: A cross-sectional survey was conducted from the 9th of January 2022 to the 2nd of February 2023, involving 7,903 physically inactive participants aged 18 to 80. Participants were recruited using a two-stage cluster sampling method from the Central Region of Saudi Arabia. In the first stage, subregions based on the administrative distribution by the Medical Service Department were selected. In the second stage, private and public entities within these subregions were identified from governmental agency lists. Participants were then conveniently approached within these entities. Data were collected using a validated questionnaire assessing sociodemographic characteristics, health history, dietary habits, and perceived internal and external barriers to physical activity.

Results: Of the participants, 67.2% were male, with a mean age of 36.45 ± 13.69 years. Approximately one-third (35%) reported experiencing at least one internal barrier to physical activity, while 64.3% reported 1–2 internal barriers. For external barriers, 76.5% faced 1–2 barriers. The most common internal barriers were laziness (40.2%) and lack of self-motivation (27.5%); the most prevalent external barriers were lack of facilities (20.2%) and long working hours (19.6%). Females were significantly more likely than males to report cultural reasons (odds ratio [OR] = 4.83; 95% confidence interval [CI]: 4.06–5.76; $p < 0.001$) and religious reasons (OR = 3.31; 95% CI: 2.59–4.23; $p < 0.001$) as internal barriers. Multivariate analysis revealed that females were 14% more likely than males to report external barriers to physical activity (OR = 1.14; 95% CI: 1.04–1.25; $p = 0.018$), suggesting gender plays a role in perceived

external obstacles. Additionally, older age, higher body mass index, higher education level, being married, certain employment statuses, and chronic diseases were significantly associated with increased reporting of internal and external barriers. These findings highlight the complex interplay of demographic and health-related factors influencing physical activity participation.

Conclusions: There is a high prevalence of both internal and external barriers to physical activity among Saudi adults, with notable gender differences influenced by cultural factors. Females were more likely to report cultural and religious reasons as barriers. Tailored policies and interventions are urgently needed to address these barriers, such as promoting gender-specific physical activity programs, integrating physical activity into workplaces, enhancing public facilities, and conducting culturally sensitive educational campaigns. Addressing both internal motivations and external obstacles is essential to increase physical activity levels and combat the rising burden of non-communicable diseases in Saudi Arabia.

Keywords: Barriers, Physical Activity, Gender Differences, Cultural Aspects, Saudi Arabia, Community Health

Introduction

Physical activity, defined by the World Health Organization (WHO) as any bodily movement produced by skeletal muscles that requires energy expenditure, is vital for maintaining health and preventing disease.[1] Regular physical activity reduces the risk of non-communicable diseases (NCDs), such as cardiovascular diseases, diabetes, and obesity, which are leading causes of mortality worldwide.[2, 3] Despite its benefits, physical inactivity remains a significant global public health issue, contributing to over 5 million deaths annually.[4]

The prevalence of physical inactivity in Saudi Arabia is particularly alarming. Studies have shown that approximately 77% of the Saudi population is physically inactive, a rate significantly higher than the global average.[5, 6] This high prevalence is attributed to rapid urbanisation, technological advancements, and lifestyle changes that have led to more sedentary behaviours.[7] Consequently, there has been a substantial increase in NCDs within the country. For instance, the prevalence of obesity among Saudi adults is estimated at around 35%, and rates of type 2 diabetes and cardiovascular diseases are on the rise.[8, 9]

Several factors contribute to physical inactivity, including age, gender, socioeconomic status, education level, and cultural influences.[10, 11] In the Saudi context, cultural and religious norms significantly impact individuals' participation in physical activity, particularly among women. Gender-specific cultural expectations often limit women's access to physical activity opportunities and facilities.[12, 13] Al-Otaibi found that cultural barriers, such as societal perceptions and lack of family support, hinder women's engagement in physical exercise in Saudi Arabia.[14]

The COVID-19 pandemic has further exacerbated physical inactivity due to lockdowns and restrictions on movement.[15] Research conducted during the pandemic indicates a decline in

physical activity levels among Saudi adults and an increase in sedentary lifestyles.[16, 17] This trend raises concerns about the long-term health implications and highlights the need for interventions that address internal and external physical activity barriers.

Understanding these barriers is crucial for developing effective strategies to promote physical activity. While previous studies have examined factors influencing physical inactivity, there is a gap in the literature regarding the combined effects of gender differences and cultural aspects, especially in the post-pandemic period. Additionally, most existing research focuses on specific populations, such as adolescents or university students, rather than a broad adult demographic. Therefore, this study aims to assess the perceived internal and external barriers to physical activity among adult residents of Riyadh, Saudi Arabia. It also seeks to identify associated sociodemographic and health-related factors, focusing on gender differences and cultural aspects. Addressing these gaps, the research intends to provide insights to inform tailored interventions and policies to enhance physical activity participation among Saudi adults.

METHODS

This cross-sectional study was conducted between January 9, 2022, and February 2, 2023. It involved 10,230 participants aged 18 to 80 from the Riyadh Region, Saudi Arabia, selected using two-staged cluster sampling. The questionnaires were distributed over 12 months across several Riyadh regions, including 8.6 million people as of the 2022 census.[18]

Sampling Technique

A two-stage cluster sampling method was employed to recruit participants:

1. **First Stage—Geographical Clustering:** The Central Region was divided into subregions based on the administrative distribution used by the Medical Service Department (MSD) of Saudi Arabia. These subregions served as the primary clusters. This stratification ensured that different geographical areas within the Central Region were adequately represented.
2. **Second Stage—Entity Selection:** Within each selected subregion, various governmental agencies obtained a list of private and public entities—including healthcare centres, workplaces, educational institutions, and public venues—which formed the secondary clusters.
3. **Participant Recruitment:** Participants were approached conveniently in each selected entity. Trained research assistants visited these locations and invited individuals to participate in the study. Convenience sampling within clusters was utilised for practical considerations and to maximise participant recruitment.

Inclusion and Exclusion Criteria

- **Inclusion Criteria:** Adults aged 18 to 80 years residing in the Central Region who were physically inactive, defined according to the WHO guidelines as not meeting the threshold of at least 150 minutes of moderate-intensity or 75 minutes of vigorous-intensity physical activity per week.[1] Physical activity was assessed through self-reporting.
- **Exclusion Criteria:** Individuals who were physically active per the above definition and those with disabilities restricting movement or preventing engagement in physical

activity were excluded. Of the initial 10,230 participants, 549 (5.4%) were identified as physically active and excluded from the study.

We reached a final number of 7903 participants, with a response rate of 81.6%. The response rate among males was higher (89.9%) than the females (60.1%), which created an imbalance in the percentage of males and females in the final sample size. The imbalance is addressed in the discussion.

The minimum accepted sample size for the 25-item questionnaire is 250. However, the sample size needed to detect logistic regression with 0.1 changes in odds ratio, power 0.9, and 0.05 error probability was 7237.[19] Therefore, the sample size is sufficient for the intended analysis.

Research tools

The tools used in this research were part of a more extensive study about physical activity and the health situation in Saudi Arabia. The participants' demographic characteristics, health history, and diet behaviour data were assessed in dedicated clinics. Four trained nurses (two males and two females) collected the data from each participant. The perceived barriers section included two subsections: the first subsection about internal barriers included 18 statements. The second subsection included external barriers, with nine statements measuring this subsection. The questionnaire tool can be described as semi-self-administrative. However, the sections measuring the barriers are answered by the participant. The validation process of the tool is described below. The validation process focused on validating the tool's inner consistency, temporal, and content validity.

Sociodemographic data

Each participant was asked to complete a sociodemographic survey, record personal and family background information, measure height and weight, and calculate Body Mass Index (BMI). $\frac{Weight\ (kg)}{Height\ (m)^2}$. Participants with BMI <18.5 were classified as underweight, 18.5-24.9 normal, 25-29.9 overweight, 30-34.9 obese class I, 35-39.9 obese class II, and ≥40 obese class III.[20] Participants were asked about their level of education, marital status, nationality (Saudi, non-Saudi), household income, employment status, smoking status, type of house where they live, daily consumption of vegetables and fruits, and medical history.

Perceived Barriers to Being Active Questionnaire (PBAQ)

The PBAQ was modified from the literature to suit the Saudi community. Questions were modified from the BBAQ 21-item questionnaire [21] and Mayo Clinic barriers to fitness.[22] Items such as cultural and religious barriers were included in the questionnaire to account for the uniqueness of Saudi Arabia's conservative and religious culture. The research team developed the additional cultural and religious items. The items were validated through two rounds with the social department at Prince Sultan Military Medical City (PSMMC). The answers to the perceived barriers to physical activity were dichotomous (apply, does not apply). The dichotomisation of the response implies information loss. Our team acknowledges this challenge. However, in the context of the sample size scale, different settings, and cultural contexts, we believe a dichotomous response comes with a reduced cognitive load compared to a 5-Likert scale, for instance ("Strongly Disagree", …, "Strongly Agree"). Also, in Saudi Arabia, discussing personal barriers may be sensitive. A simple "apply" or "does not apply" format might encourage more honest and straightforward responses. Dichotomous answers can enhance the

reliability of the responses, as they eliminate ambiguity inherent in scaled responses. The validation process showed that participants were more consistent or comfortable with dichotomous responses during pilot testing. This supports our choice. While using scales can provide more nuanced data, it also introduces complexity in statistical analysis, especially when ensuring the validity and reliability of the scales across different items and constructs. Since our study aims to identify whether specific barriers exist, dichotomous responses directly address this research question without unnecessary complexity.

Validation of PBAQ

The questionnaire was judged to have content validity as the items adopted from validated questionnaires except for the items the team developed. Therefore, we contacted three social consultants at PSMMC and the Medical Service Department (MSD) to provide their feedback. The three consultants are part of the integrated care the Chronic Disease Department provides at PSMMC. These consultants are exposed to tailoring different programmes to accommodate patients' social contexts. Content Validity Index (CVI) was produced for the tool. The content validity process was considered complete when the consultants perceived all the items as relevant in the tool through the CVI. Three physicians conducted face validity for the Family and Community Medicine Department questionnaire in the PSMMC hospital. A linguist from King Saud University in Riyadh translated and verified the Arabic version of the questionnaire. A professional Arabic-English translator at the hospital retranslated the Arabic version to English. We compared the original and retranslated versions. Significant differences were discussed with the two translators separately. The process needed three rounds of translation until the translators agreed on a final version.

The research team conducted a test-retest to assess the reliability of the research tool. A convenient sample of 361 physically inactive participants from the staff of the PSMMC and the Al-Wazarat primary health care centre in Riyadh were asked to complete the questionnaire. The participants were informed if they were willing to answer the questionnaire again; 318 participants expressed their willingness to answer the questionnaire a second time. The research team distributed the questionnaire again two weeks after the initial distribution. The average time between the initial and second distribution was 15.6 days. The questionnaire's inner consistency was assessed using Cronbach's alpha = 0.83. The questionnaire had 64%-89% intraclass correlation coefficients (ICC) and 68%-91% temporal stability. The results of the test-retest questionnaire were acceptable for the research tool to be considered reliable and distributed by the research team.

Statistical methods

Data were analysed using R version 4.0.[23] In addition to descriptive statistics and data visualisations, we calculated odds ratios and 95% confidence intervals (CIs) to compare males and females. We used the male group as a reference group for all calculation comparisons. The association between the participants' characteristics and the barriers was investigated through logistic regression. Two relations were modelled, one for internal barriers and the other for external barriers. We dichotomised the answers to 0 = no barriers reported and 1 = at least one barrier reported. We believe modelling two separate models for internal and external barriers is more meaningful. The two sets of barriers may vary differently among the participants. For instance, some participants may report they have no internal barriers, but external barriers prevent them from being physically active, and vice versa.

We applied the logistic regression to predict the barriers to physical activity. The best-fitting model selection is based on information criteria. We used Akaike's Information Criterion (AIC) and Bayesian Information Criterion (BIC) as selection criteria. AIC is more liberal than BIC in penalising complexity. However, AIC is more theoretically established than BIC.[24] The model selection process was conducted using the AIC and BIC stepwise approach. Additionally, multicollinearity using variance inflated factor (VIF) and logistic regression assumptions were checked pre- and post-analysis. Confounding variables were adjusted for in the models based on the studies by Lau et al.,[25] O'Donoghue et al.,[26] and Bauman et al.[27] The full model included all variables collected from everyone. After checking multicollinearity and using stepwise AIC and BIC, the adjusted model was used as a final model. We assumed that the independence assumption of the observations was satisfied due to the randomness of the sample.

Ethical Considerations

The Institutional Review Board (IRB) at PSMMC approved the study. Before data collection, all participants provided written informed consent. For illiterate participants or those with visual impairments, a trained nurse read the questionnaire aloud, and verbal consent was obtained in the presence of a witness. Participant confidentiality and anonymity were strictly maintained throughout the study.

RESULTS

Sociodemographic characteristics of participants

The first section of the questionnaire concerned each participant's sociodemographic and background data. Table 1 shows the results of the distributed questionnaires. Male participants

formed most respondents, 5313 (67.2%). In both gender groups, most of the participants were younger than 45 years old. The age categories with the most participants are 26-35 (31.4%), 18-25 (24.4%), and 36-45 (21.5%). Most of the participants were Saudi nationals (67.8). BMI measurements showed an average of 25.88 (± 3.9), with no difference between males and females. However, over half (55.4%) of participants were overweight or obese. Participants were normally distributed between categories for the level of education, with more than three-quarters of them having secondary or college education. Also, three-quarters of the participants were married, and more than half worked full-time. 1 out of 5 participants was a smoker, and almost 1 out of 2 participants did not consume fruits and vegetables.

The health status of the participants

Table 2 shows the distribution of participants over the different non-communicable diseases and injuries. Musculoskeletal disorders significantly differed among all screened diseases between males and females with OR 1.39 (1.15, 1.69, p-value <0.001). CVD and other ailments have high OR, 1.37 (0.93, 1.99, p-value = 0.063) and 1.13 (0.54, 2.36, p-value = 0.440), respectively. However, they were not significantly different, i.e., p-value ≥ 0.05. Diabetes, with 20.9% of the participants, was the most prevalent disease in the sample.

Internal barriers to physical activity

Figure 1b illustrates that males and females report comparable internal barriers to physical activity. By analysing the percentage distribution of reported barriers, we account for the sample size imbalance between genders, allowing for a more accurate comparison. As shown in Figure 1c, most participants reported two (35%) or one (29.3%) internal barriers.

Table 3 lists the internal barriers to physical activity according to gender in Saudi Arabia. The internal barriers were grouped into the following categories: lack of energy, motivation, self-efficacy, and beliefs and thoughts. For the first category, males and females expressed that laziness (OR = 0.71, 95% CI (0.65, 0.79), p-value < 0.001) and boredom (OR = 0.78. 95% CI (0.67, 0.92), p-value = 0.033) were reasons to lower their exercise energy. Being a male was significantly higher, as the OR < 1 and CI show.

Lacking motivation is considered a barrier to sustaining an activity. Self-motivation, encouragement from others, and knowledge about physical activities are components of such motivation. Females were significantly less motivated to engage in physical activity and more affected by encouragement from others. Self-efficacy, including self-confidence, self-management, and physical abilities, was another common barrier for males and females.

Moreover, when exercising around others, females expressed embarrassment about their bodies OR = 2.44 and 95% CI (1.84, 3.23), p-value <0.001. Finally, barriers due to beliefs and thoughts for cultural or religious reasons were evident in the case of female participants. For cultural and religious reasons, the highest odd ratios between males and females regarding internal barriers were in this category. Females were more likely with OR = 3.31, 95% CI (2.59, 4.23), and p-value <0.001 to report religious reasons as a barrier for physical activity, also were more likely with OR = 4.83, 95% CI (4.06, 5.76), and p-value < 0.001 to report cultural reasons as a barrier for physical activity.

Number of internal barriers to physical activity amongst Saudi adults

While 1 out of every 5 Saudi adults expressed three barriers or more, most participants (64.3%) had 1-2 internal barriers. Only 12.8% and 14.1% of females and males declared no internal

barriers to engaging in physical activity, and females showed higher percentages as the number of barriers increased.

External barriers to physical activity

Disparities between males and females are evident in the number of external barriers reported, mainly when participants report more than three barriers. As shown in Figure 2b, 16% of female participants reported three external barriers, compared to 10% of male participants. This indicates that females are 54% more likely than males to report three barriers [(16% - 10.4%) / 10.4% = 54%]. Additionally, 2.4% of females reported four or more external barriers, compared to 0.8% of males, meaning females are 200% more likely than males to report four or more barriers [(2.4% - 0.8%) / 0.8% = 200%]. Like internal barriers, reporting one barrier (34.3%) or two (42.2%) was most common among participants.

Table 4 lists the external barriers to physical activity according to gender in Saudi Arabia. The external barriers were grouped into the following categories: lack of facilities, lack of time and other reasons. Around 20% of the participants considered the unavailability of facilities a barrier to the exercise. Females showed higher percentages than males with OR = 2.25, CI (2.02, 2.50), and p-value < 0.001 for gym or facility availability. On the other hand, only around 15% of the participants considered the weather as a barrier. For working long hours, having night shifts and travelling, males showed higher percentages and more male-specific barriers. Nevertheless, family obligations and commitments were female-specific barriers with OR = 4.2, CI (3.76, 4.70), and p-value < 0.001.

Other external barriers, in addition to those mentioned, may play a role in lowering engagement in physical activities. However, they were not specified in this study. The barriers were found to be similar in both male and female participants.

Number of external barriers to physical activity amongst Saudi adults

Only 8% of females and 11% of males declared no external barriers to physical activity; this result is less than the participants who reported no internal barriers. Most participants (76.5%) had 1-2 external barriers, while the rest had three or more external ones. Also, females showed higher percentages as the number of external barriers increased.

Association between internal barriers with sociodemographic status

The logistic regression model associated sociodemographic status with internal barriers (Table 5). We predicted that a Saudi adult who is overweight or obese, older age, married (or has been married), highly educated, retired, a smoker (or ex-smoker), and has chronic diseases was at high risk of physical inactivity regardless of gender. However, household income was not associated with internal barriers.

Association between external barriers with sociodemographic status

External barriers were associated with higher education, being married (or having been married), having many family members, being a student, and not consuming fruits and vegetables daily (Table 6).

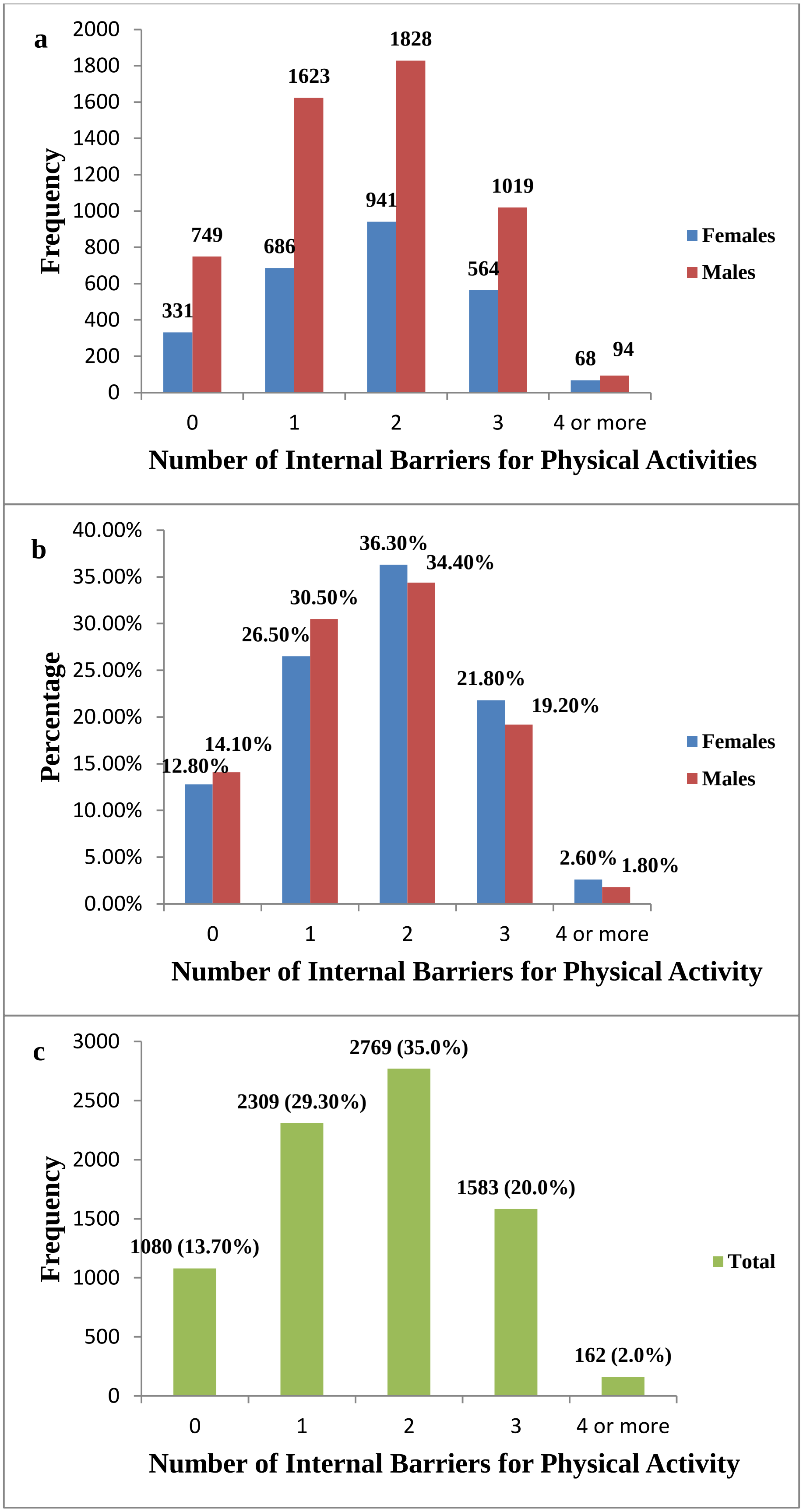
a
Frequency
2000
1800
1600
1400
1200
1000
800
600
400
200
0
331
749
686
1623
941
1828
564
1019
68
94
0
1
2
3
4 or more
Number of Internal Barriers for Physical Activities
Females
Males
b
Percentage
40.00%
35.00%
30.00%
25.00%
20.00%
15.00%
10.00%
5.00%
0.00%
12.80%
14.10%
26.50%
30.50%
36.30%
34.40%
21.80%
19.20%
2.60%
1.80%
0
1
2
3
4 or more
Number of Internal Barriers for Physical Activity
Females
Males
c
Frequency
3000
2500
2000
1500
1000
500
0
1080 (13.70%)
2309 (29.30%)
2769 (35.0%)
1583 (20.0%)
162 (2.0%)
0
1
2
3
4 or more
Number of Internal Barriers for Physical Activity
Total

Figure 1: a) Frequencies of the number of internal barriers chosen by participants stratified by gender. b) Percentages for the number of internal barriers to physical activity chosen by the participants stratified by gender. c) Frequencies and percentages of the number of internal barriers chosen by participants (all)

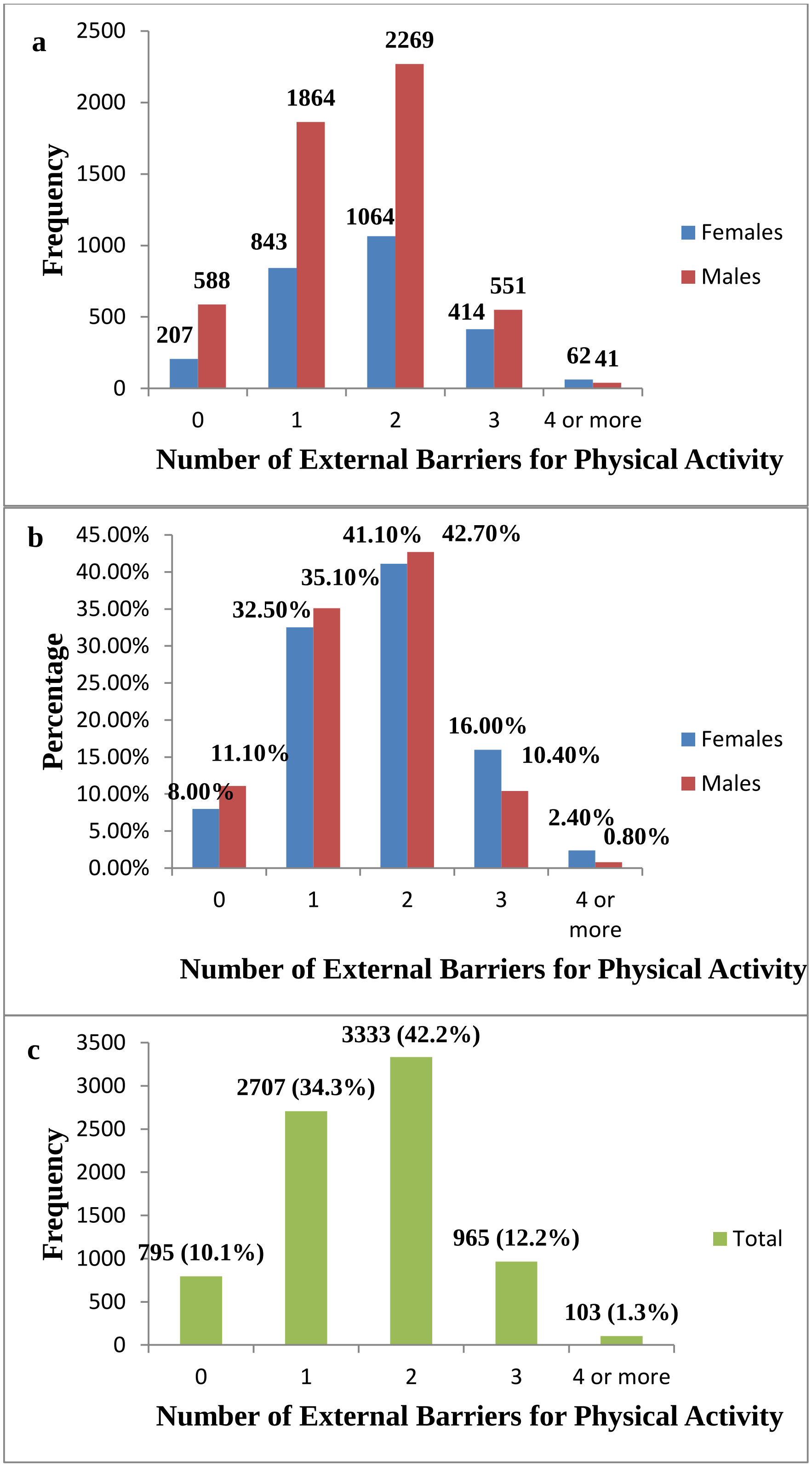

Figure 2: a) Frequencies of the number of external barriers chosen by participants stratified by gender. b) Percentages for the number of external barriers to physical activity chosen by the participants stratified by gender. c) Frequencies and percentages of the number of external barriers chosen by participants (all).

Table 1: Participants' sociodemographic characteristics stratified by gender (n=7903)

Characteristic	Female (%)	Male (%)	Total (%)	χ^2 P-value[a]
**Age (years)**	36.46 ± 13.54[b]	36.44 ± 13.84[b]	36.45 ± 13.69[b]	0.006
18-25	617 (23.8)	1308 (24.6)	1925 (24.4)	
26-35	841 (32.5)	1637 (30.8)	2478 (31.4)	
36-45	553 (21.4)	1146 (21.6)	1699 (21.5)	
46-55	231 (8.9)	463 (8.7)	694 (8.8)	
56-65	290 (11.2)	555 (10.4)	845 (10.7)	
>65	58 (2.2)	204 (3.8)	262 (3.3)	
**Total**	**2590 (100)**	**5313 (100)**	**7903 (100)**	
**Nationality**				<0.001
Saudi	1603 (61.9)	3755 (70.7)	5358 (67.8)	
Non-Saudi	987 (38.1)	1558 (29.3)	2545 (32.2)	
**Total**	**2590 (100)**	**5313 (100)**	**7903 (100)**	
**BMI**[c]	25.91 ± 3.93[b]	25.88 ± 3.90[b]	25.80 ± 3.92[b]	0.001
Underweight	38 (1.5)	94 (1.8)	132 (1.7)	
Normal	1042 (40.2)	2350 (44.2)	3392 (42.9)	
Overweight	1245 (48.1)	2296 (43.2)	3541 (44.8)	
Obese Class I	192 (7.4)	423 (8.0)	615 (7.8)	
Obese Class II	55 (2.1)	125 (2.4)	180 (2.3)	
Obese Class III	19 (0.7)	25 (0.5)	43 (0.5)	
**Total**	**2590 (100)**	**5313 (100)**	**7903 (100)**	
**Education**				<0.001
No Education	110 (4.2)	178 (3.4)	288 (3.6)	
Elementary School	324 (12.5)	603 (11.3)	927 (11.7)	
Secondary School	1100 (42.5)	2218 (41.7)	3318 (42.0)	
First College or University Degree	856 (33.1)	1931 (36.3)	2787 (35.3)	
Higher Education	200 (7.7)	383 (7.2)	583 (7.4)	
**Total**	**2590 (100)**	**5313 (100)**	**7903 (100)**	
**Marital status**				<0.001
Single	435 (16.8)	1232 (23.2)	1667 (21.1)	
Married	2097 (81.0)	3973 (74.8)	6070 (76.8)	
Divorced/Separated	50 (1.9)	75 (1.4)	125 (1.6)	
Widowed	8 (0.3)	33 (0.6)	41 (0.5)	
**Total**	**2590 (100)**	**5313 (100)**	**7903 (100)**	
**Household income (SAR/Month)**[d]				0.720
<3,000	148 (5.7)	319 (6.0)	467 (5.9)	
3,000 – 6,000	1033 (39.9)	2094 (39.4)	3127 (39.6)	
6,001 – 9,000	668 (25.8)	1332 (25.1)	2000 (25.3)	
9,001 – 12,000	344 (13.3)	695 (13.1)	1039 (13.1)	
> 12,000	397 (15.3)	873 (16.4)	1270 (16.1)	
**Total**	**2590 (100)**	**5313 (100)**	**7903 (100)**	
**Number of household members**				0.006
1 – 2	102 (3.9)	213 (4.0)	315 (4.0)	
3 – 5	1042 (40.2)	2357 (44.4)	3399 (43.0)	
6 – 7	1286 (49.7)	2472 (46.5)	3758 (47.6)	
7 – 9	127 (4.9)	220 (4.1)	347 (4.4)	
>9	33 (1.3)	51 (1.0)	84 (1.1)	
**Total**	**2590 (100)**	**5313 (100)**	**7903 (100)**	

**Employment Status**				<0.001
Full-time (>=30 hours/week)	721 (27.8)	3746 (70.5)	4467 (56.5)	
Part-time (<30 hours/week)	182 (7.0)	260 (4.9)	442 (5.6)	
Unemployed (actively looking)	137 (5.3)	190 (3.6)	327 (4.1)	
Unemployed (not looking and healthy)	1048 (40.5)	61 (1.1)	1109 (14.0)	
Unemployed (not looking for health reasons)	2 (0.1)	34 (0.6)	36 (0.5)	
Student	221 (8.5)	472 (8.9)	693 (8.8)	
Retired	279 (10.8)	550 (10.4)	829 (10.5)	
**Total**	**2590 (100)**	**5313 (100)**	**7903 (100)**	
**Smoking status**[a]				<0.001
Non-Smoker	2361 (91.2)	3713 (69.9)	6074 (76.9)	
Smoker	199 (7.7)	1476 (27.8)	1675 (21.2)	
Ex-Smoker	30 (1.2)	124 (2.3)	154 (1.9)	
**Total**	**2590 (100)**	**5313 (100)**	**7903 (100)**	
**House type**				0.617
Detached house with a yard	127 (4.9)	294 (5.5)	421 (5.3)	
A detached house without a yard	425 (16.4)	850 (16.0)	1257 (16.1)	
Apartment with roof or yard	75 (2.9)	175 (3.3)	250 (3.2)	
Apartment with no roof or yard	1954 (75.4)	3972 (74.8)	5926 (75.0)	
Others	9 (0.3)	22 (0.4)	31 (0.4)	
**Total**	**2590 (100)**	**5313 (100)**	**7903 (100)**	
**Daily consumption of vegetables and fruits**				0.012
Yes	1125 (43.4)	2467 (46.4)	3592 (45.5)	
No	1465 (56.6)	2846 (53.6)	4311 (54.5)	
**Total**	**2590 (100)**	**5313 (100)**	**7903 (100)**	

[a] P-value based on the Chi-squared test of association is considered significant if <0.05

[b] Mean ± Standard deviation

[c] BMI classification (underweight <18.5, Normal 18.5 – 24.9, Overweight 25 – 29.9, Obese Class I 30 – 34.9, Obese Class II 35 – 39.9, Obese Class III 40 and above)

[d] Income is expressed in Saudi Riyal (SAR), the exchange against the US Dollar is fixed at 1 USD ≈ 3.75 SAR

[e] smoking includes smoking cigarettes, vapes, electronic cigarettes, and water pipes (hookah)

Table 2: Diseases and injuries in the sample stratified by the participants' gender (n=7903)

Diseases or injuries	Female (%)	Male (%)	Total (%)	OR[b] (95% Confidence Interval)	χ^2 P-value[a]
CVD[c]					
Present	45 (1.7)	68 (1.3)	113 (1.3)	1.37 (0.93, 1.99)	0.067
Absent	2545 (98.3)	5245 (98.7)	7790 (98.6)		
**Total**	**2590 (100)**	**5313 (100)**	**7903 (100)**		
Brain and neurological diseases or injuries					
Present	4 (0.2)	11 (0.2)	15 (0.2)	0.74 (0.24, 2.34)	0.422
Absent	2586 (99.8)	5302 (99.8)	7888 (99.8)		
**Total**	**2590 (100)**	**5313 (100)**	**7903 (100)**		
Musculoskeletal disorders					
Present	184 (7.1)	277 (5.2)	461 (5.8)	1.39 (1.15, 1.69)	0.001
Absent	2406 (92.9)	5036 (94.8)	7442 (94.2)		
**Total**	**2590 (100)**	**5313 (100)**	**7903 (100)**		
Cancer					
Present	15 (0.6)	33 (0.6)	48 (0.6)	0.93 (0.50, 1.72)	0.479
Absent	2575 (99.4)	5280 (99.4)	7855 (99.4)		
**Total**	**2590 (100)**	**5313 (100)**	**7903 (100)**		
Diabetes					
Present	520 (20.0)	1131 (21.3)	1651 (20.9)	0.94 (0.84, 1.06)	0.214
Absent	2070 (80.0)	4182 (78.7)	6252 (79.1)		
**Total**	**2590 (100)**	**5313 (100)**	**7903 (100)**		
Chronic Kidney Disease					
Present	32 (1.2)	76 (1.4)	108 (1.4)	0.86 (0.57, 1.31)	0.278
Absent	2558 (98.8)	5237 (98.6)	7795 (98.6)		
**Total**	**2590 (100)**	**5313 (100)**	**7903 (100)**		
Other diseases					
Present	11 (0.4)	20 (0.4)	31 (0.4)	1.13 (0.54, 2.36)	0.440
Absent	2579 (99.6)	5293 (99.6)	7872 (99.6)		
**Total**	**2590 (100)**	**5313 (100)**	**7903 (100)**		

[a] Chi-squared test of association is considered statistically significant if the p-value is less than 0.05
[b] OR: Odds ratio (Male group is the reference group)
[c] CVD: Cardiovascular Diseases

Table 3: The distribution of internal barriers to physical activity according to gender in Saudi Arabia (n=7903)

Barrier	Female (n=2590)		Male (n=5313)		χ^2 P-value[a]	OR[b] (95% Confidence Interval)
	Yes (%)	No (%)	Yes (%)	No (%)		
**lack of energy**						
I am too lazy	943 (36.41)	1647 (63.59)	2366 (44.53)	2947 (55.47)	<0.001*	0.71 (0.65, 0.79)
I find it inconvenient to exercise	61 (2.36)	2529 (97.64)	93 (1.75)	5220 (98.25)	0.068	1.35 (0.98, 1.88)
I find exercise boring	231 (8.92)	2359 (91.08)	588 (11.07)	4725 (88.93)	0.033*	0.78 (0.67, 0.92)
**Lack of motivation**						
Lack self-motivation	728 (28.11)	1862 (71.98)	1421 (26.75)	3892 (73.25)	0.202	1.07 (0.96, 1.18)
I lack encouragement, support, or companionship from family and friends	247 (9.54)	2343 (90.46)	322 (6.06)	4991 (93.94)	<0.001*	1.63 (1.37, 1.94)
**lack of self-efficacy**						
I am embarrassed and self-conscious about my body. I do not like to exercise around other people	108 (4.17)	2482 (95.83)	93 (1.75)	5220 (98.25)	<0.001*	2.44 (1.84, 3.23)
I lack self-management skills	536 (20.69)	2054 (79.31)	925 (17.41)	4388 (82.59)	<0.001*	1.24 (1.10, 1.39)
I do not know how to be physically active	94 (3.63)	2496 (96.37)	141 (2.65)	5172 (97.35)	0.016*	1.38 (1.06, 1.80)
I lack confidence in my ability to be physically active	352 (13.59)	2238 (86.41)	764 (14.38)	4549 (85.62)	0.345	0.93 (0.81, 1.07)
I feel too old to do exercises	34 (1.31)	2556 (98.69)	86 (1.62)	5227 (98.38)	0.300	0.81 (0.54, 1.21)
I do not have the skills required to do sports	211 (8.15)	2379 (91.85)	597 (11.24)	4716 (88.76)	<0.001*	0.70 (0.59, 0.83)
I fear being injured or have been injured recently	216 (8.34)	2374 (91.66)	628 (11.82)	4685 (88.18)	<0.001*	0.68 (0.58, 0.80)
I have a health condition that does not allow me to do physical activities	170 (6.56)	2420 (93.44)	336 (6.32)	4977 (93.68)	0.680	1.04 (0.86, 1.26)
**Beliefs and thoughts**						
I do not do it for religious reasons	168 (6.49)	2422 (93.51)	109 (2.05)	5204 (97.59)	<0.001*	3.31 (2.59, 4.23)
It is not a cultural thing, so I do not do it	421 (16.25)	2169 (83.75)	205 (3.86)	5108 (96.14)	<0.001*	4.83 (4.06, 5.76)
I do not think it is beneficial for the health	12 (0.46)	2578(99.54)	48 (0.9)	5265 (99.10)	0.034*	0.51 (0.27, 0.96)

[a] Chi-squared test of association is considered statistically significant if the p-value is less than 0.05

[b] OR: Odds Ratios (Males group is the reference group)

* p-value is statistically significant at 0.05 level of significance

Table 4: The distribution of external barriers to physical activity according to gender in Saudi Arabia (n=7903)

Barrier	Female (n=2590)		Male (n=5313)		χ^2 P-value[a]	OR[b] (95% Confidence Interval)
	Yes (%)	No (%)	Yes (%)	No (%)		
**Lack of facilities**						
I do not have the means to access a sporting facility (e.g. no car, too expensive)	511 (20)	2079 (80)	738 (14)	4575 (86)	<0.001*	1.52 (1.35, 1.73)
There are no facilities near my residence (gyms, parks, or safe places for walking)	890 (34)	1700 (66)	1003 (19)	4310 (81)	<0.001*	2.25 (2.02, 2.50)
Mostly the weather is not convenient for walking or doing physical activity	332 (13)	2258 (87)	812 (15)	4501 (85)	0.003*	0.82 (0.71, 0.94)
**Lack of time**						
I work night shifts, and it is affecting my ability to do physical activities	106 (4)	2484 (96)	671 (13)	4642 (87)	<0.001*	0.30 (0.24, 0.36)
My working hours are too long. I cannot do extra activities during the day	151 (6)	2493 (94)	1398 (26)	3915 (74)	<0.001*	0.17 (0.14, 0.20)
My work requires travelling a lot, and I cannot commit	14 (1)	2576 (99)	113 (2)	5200 (98)	<0.001*	0.25 (0.14, 0.43)
Family obligations I do not find enough time and energy myself to do physical activity	2094 (81)	496 (19)	2663 (50)	2650 (50)	<0.001*	4.20 (3.76, 4.70)
**Other Barriers**						
My job is so demanding physically I feel exhausted afterwards	281 (11)	2309 (89)	724 (14)	4589 (86)	<0.001*	0.77 (0.67, 0.89)
Other reasons	46 (2)	2544 (98)	97 (2)	5216 (98)	0.888	0.97 (0.68, 1.39)

[a] Chi-squared test of association is considered statistically significant if the p-value is less than 0.05
[b] OR: Odds Ratios (Males group is the reference group)
* p-value is statistically significant at a 0.05 level of significance

Table 5: The best-fitting logistic regression investigating the correlation between the participants' characteristics and the presence of internal barriers to physical activity (n= 7903)

Characteristic*	Odds Ratio	P-value	95% Confidence Interval of Odds Ratio
**Age (years)**	1.02	0.009	[1.1,1.3]
**Nationality**			
**Saudi**	Reference	Reference	Reference
**Non-Saudi**	0.88	0.002	[0.81, 0.96]
**BMI****[**]**	1.16	<0.001	[1.12, 1.35]
**Education**			
**No Education**	Reference	Reference	Reference
**Elementary School**	0.86	0.008	[0.81, 0.93]
**Secondary School**	0.81	<0.001	[0.73, 0.87]
**First College or University Degree**	1.10	0.028	[1.06, 1.34]
**Higher Education**	1.61	<0.001	[1.23, 2.85]
**Marital status**			
**Single**	Reference	Reference	Reference
**Married**	2.71	<0.001	[1.39, 4.61]
**Divorced/Separated**	1.24	0.056	[0.98, 2.18]
**Widowed**	2.26	0.042	[1.04, 5.87]
**Household income (SAR/Month)**[a]			
**<3,000**	Reference	Reference	Reference
**3,000 – 6,000**	1.08	0.772	[0.62, 2.71]
**6,001 – 9,000**	0.84	0.036	[0.79, 0.92]
**9,001 – 12,000**	0.89	0.048	[0.83, 0.99]
**> 12,000**	0.95	0.311	[0.72, 1.66]
**Employment Status**			
**Full-time (>=30 hours/week)**	Reference	Reference	Reference
**Part-time (<30 hours/week)**	0.91	0.282	[0.87, 1.18]
**Unemployed (actively looking)**	1.03	0.690	[0.57, 2.73]
**Unemployed (not looking and healthy)**	0.65	<0.001	[0.42, 0.79]
**Unemployed (not looking for health reasons)**	3.04	<0.001	[1.51, 5.92]
**Student**	0.77	<0.001	[0.61, 0.89]
**Retired**	6.55	<0.001	[3.63, 11.76]
**Smoking status**[b]			
**Non-Smoker**	Reference	Reference	Reference
**Smoker**	1.98	<0.001	[1.27, 3.08]
**Ex-Smoker**	2.62	<0.001	[1.49, 6.11]

**Chronic diseases comorbidities**			
**Yes**	Reference	Reference	Reference
**No**	0.29	<0.001	[0.08, 0.41]

* The best-fitting model with lowest AIC = 768.8 and BIC = 811.2. The initial model included Gender, Age, Nationality, Body Mass Index (BMI), Education, Marital Status, Household Income, Number of Household Members, Employment Status, Smoking Status, House Type, Presence of Chronic Comorbidities, Daily Consumption of Vegetables and Fruits.

** BMI: Body Mass Index: for every five units increase in BMI

[a] Income is expressed in Saudi Riyal (SAR), and the exchange against the US Dollar is fixed at 1 USD ≈ 3.75 SAR

[b] smoking includes smoking cigarettes, vapes, electronic cigarettes, and water pipes (hookah)

Table 6: The best-fitting logistic regression investigating the correlation between the participants' characteristics and the presence of external barriers to physical activity (n= 7903)

Characteristic	Odds Ratio	P-value	95% Confidence Interval of Odds Ratio
**Gender**			
**Male**	Reference	Reference	Reference
**Female**	1.14	0.018	[1.04, 1.25]
**Age (years)**	1.01	0.046	[1.00, 1.02]
**BMI****	1.18	<0.001	[1.15, 1.41]
**Nationality**			
**Saudi**	Reference	Reference	Reference
**Non-Saudi**	0.93	0.039	[0.86, 0.97]
**Education**			
**No Education**	Reference	Reference	Reference
**Elementary School**	0.97	0.073	[0.95, 1.22]
**Secondary School**	0.89	0.460	[0.63, 2.18]
**First College or University Degree**	2.21	<0.001	[1.56, 3.89]
**Higher Education**	3.39	<0.001	[1.61, 9.27]
**Marital status**			
**Single**	Reference	Reference	Reference
**Married**	4.09	<0.001	[1.91, 13.06]
**Divorced/Separated**	2.55	<0.001	[1.52, 6.32]
**Widowed**	1.34	0.601	[0.34, 3.51]
**Household income (SAR/Month)**[d]			
**<3,000**	Reference	Reference	Reference
**3,000 – 6,000**	0.96	0.106	[0.91, 1.02]
**6,001 – 9,000**	1.08	0.533	[0.89, 1.61]
**9,001 – 12,000**	0.86	0.011	[0.78, 0.94]
**> 12,000**	0.73	<0.001	[0.61, 0.83]
**Number of household members**			
**1 – 2**	Reference	Reference	Reference
**3 – 5**	1.13	0.229	[0.92, 1.46]
**6 – 7**	1.69	0.059	[0.98, 1.91]
**7 – 9**	2.06	<0.001	[1.21, 3.14]
**>9**	4.30	<0.001	[2.12, 6.86]

**Employment Status**			
**Full-time (>=30 hours/week)**	Reference	Reference	Reference
**Part-time (<30 hours/week)**	1.09	0.319	[0.88, 1.75]
**Unemployed (actively looking)**	1.62	0.099	[0.79, 2.01]
**Unemployed (not looking and healthy)**	0.66	0.006	[0.40, 0.96]
**Unemployed (not looking for health reasons)**	0.38	<0.001	[0.18, 0.54]
**Student**	1.88	<0.001	[1.14, 3.10]
**Retired**	0.72	0.582	[0.28, 1.61]
**Daily consumption of vegetables and fruits**			
**Yes**	Reference	Reference	Reference
**No**	1.93	<0.001	[1.22, 3.10]

* The best-fitting model with lowest AIC = 359.7 and BIC = 382.4. The initial model included Gender, Age, Nationality, Body Mass Index (BMI), Education, Marital Status, Household Income, Number of Household Members, Employment Status, Smoking Status, House Type, Presence of Chronic Comorbidities, Daily Consumption of Vegetables and Fruits.

** BMI: Body Mass Index, every five units increase in BMI

[a] Income is expressed in Saudi Riyal (SAR), and the exchange against the US Dollar is fixed at 1 USD ≈ 3.75 SAR

[b] smoking includes smoking cigarettes, vapes, electronic cigarettes, and water pipes (hookah)

DISCUSSION

Summary of Findings

This large cross-sectional study assessed the perceived barriers to physical activity among 7,903 physically inactive Saudi adults aged 18 to 80 in the Central Region of Saudi Arabia. The findings revealed that internal and external barriers are prevalent, with notable gender differences influenced by cultural factors. Approximately one-third of participants reported experiencing at least one internal barrier, and 64.3% reported one or two internal barriers. Laziness (40.2%) and lack of self-motivation (27.5%) were the most common internal barriers. For external barriers, 76.5% faced one or two, with the lack of facilities (20.2%) and long working hours (19.6%) being the most prevalent. Females were significantly more likely than males to report cultural (odds ratio [OR] = 4.83; 95% confidence interval [CI]: 4.06–5.76; $p < 0.001$) and religious reasons (OR = 3.31; 95% CI: 2.59–4.23; $p < 0.001$) as internal barriers. Multivariate analyses indicated that gender, age, higher body mass index (BMI), education level, marital status, employment status, and chronic diseases were significantly associated with reporting internal and external barriers.

Comparison with Previous Studies

Our findings align with previous research indicating that females face more barriers to physical activity than males, particularly in conservative societies.[28-30] Cultural and religious norms in Saudi Arabia often limit women's participation in physical activities due to societal expectations and restrictions on gender interactions.[8, 31] Similar barriers have been reported in other

Muslim-majority or with large Muslim community countries, such as Malaysia and India, where cultural expectations and lack of social support hinder women's engagement in physical activity.[32, 33]

Age was positively associated with reporting barriers, consistent with studies showing that physical activity tends to decrease due to decreased motivation, health issues, and limited mobility.[34, 35] Older individuals may face additional internal barriers, such as fear of injury and lack of confidence, as well as external barriers, like inadequate facilities suitable for their age group.

Higher BMI was associated with increased reporting of internal and external barriers. This relationship reflects the bidirectional link between obesity and physical inactivity, where higher BMI may result from inactivity. Conversely, individuals with higher BMI may find it more challenging to engage in physical activity due to physical discomfort or self-consciousness.[36] Similar associations have been observed in other studies.[37]

Contrary to expectations, higher education levels were associated with more reported barriers to physical activity. While education is generally associated with greater health awareness, our findings are consistent with research from developing countries where higher education correlates with sedentary occupations and time constraints.[37-39] In contrast, studies from developed countries often show that higher education levels are linked to increased physical activity, possibly due to greater access to resources and recreational facilities.[39-41] This discrepancy may be influenced by cultural and socioeconomic factors unique to different regions.

Marital status and family size significantly impacted barrier reporting. Married individuals and those with larger households reported more internal and external barriers. This may be due to increased family responsibilities, particularly among women prioritising household obligations over personal activities.[33, 42] Similar findings have been reported in other studies where family commitments limit the time available for physical activity.[42]

Employment status influenced perceived barriers, with retirees reporting more internal barriers. This could be attributed to age-related factors and potential health issues accompanying retirement age. However, full-time employment was also associated with external barriers such as long working hours and lack of time, consistent with findings from other research indicating that occupational demands limit opportunities for physical activity.[43, 44]

Implications for Practice and Policy

The high prevalence of internal and external barriers underscores the need for comprehensive strategies to promote physical activity among Saudi adults. Gender-specific interventions are crucial, considering the significant cultural and religious barriers faced by women. Potential strategies include:

- **Improving Access to Facilities:** Developing gender-specific and culturally appropriate physical activity facilities, such as women-only gyms and safe public spaces, can help reduce external barriers to facility availability.
- **Workplace Interventions:** Implementing workplace wellness programs incorporating physical activity can address time constraints due to long working hours, benefiting both male and female employees.

- **Culturally Sensitive Education Campaigns:** Designing educational initiatives that respect cultural and religious values while promoting the health benefits of physical activity can help overcome internal barriers, particularly among women.
- **Community Engagement:** Encouraging community-based physical activities and leveraging social support networks can enhance motivation and reduce barriers to lack of encouragement or companionship.
- **Policy Development:** Policymakers should consider integrating physical activity promotion into national health strategies, addressing infrastructural limitations, and supporting initiatives facilitating active lifestyles.

Limitations

Despite the significant findings, this study has several limitations:

- **Gender Imbalance:** The sample consisted predominantly of male participants (67.2%), which may limit the generalizability of the results, particularly concerning women's barriers to physical activity. Cultural norms restricting women's participation in public activities may have contributed to this imbalance.
- **Regional Representation:** The study was conducted in the Central Region of Saudi Arabia, which may not represent other regions' diverse cultural and socioeconomic contexts. Therefore, the findings may not be generalised to the entire Saudi adult population.
- **Sampling Method:** Using convenience sampling within clusters could introduce selection bias, affecting the sample's representativeness. Participants who are more accessible or willing to participate may differ systematically from those who are not.

- **Self-Reported Data:** Reliance on self-reported measures for physical activity levels and perceived barriers may lead to recall or social desirability bias. Participants might underreport or overreport certain behaviours or barriers.
- **Cross-Sectional Design:** The study's cross-sectional nature limits the ability to establish causality between sociodemographic factors and perceived barriers. Longitudinal studies are needed to understand temporal relationships and causal pathways.

Recommendations for Future Research

Future studies should aim to:

- **Enhance Gender Representation:** Employ strategies to increase female participation, such as utilising female data collectors or creating culturally appropriate recruitment methods.
- **Expand Regional Coverage:** To improve the generalizability of the findings, include participants from various regions across Saudi Arabia.
- **Use Objective Measures:** Incorporate objective physical activity assessments like accelerometers to complement self-reported data and reduce bias.
- **Longitudinal Studies:** Conduct longitudinal research to explore causal relationships and evaluate the effectiveness of interventions over time.
- **Qualitative Research:** Utilize qualitative methods to gain deeper insights into the specific cultural and religious factors influencing physical activity behaviours, particularly among women.

Conclusion

This study highlights the multifaceted barriers to physical activity among Saudi adults, with significant gender differences shaped by cultural and religious factors. Addressing these barriers requires tailored, culturally sensitive interventions that consider Saudi Arabia's unique societal context. By implementing targeted strategies and policies, it is possible to enhance physical activity levels, thereby reducing the burden of non-communicable diseases and improving the overall health and well-being of the population.

Declaration of interest conflict

The authors declare no conflict of interest.

References

1. World Health Organization. WHO Guidelines on Physical Activity and Sedentary Behaviour Geneva: WHO; 2021 [Available from: https://www.who.int/multi-media/details/who-guidelines-on-physical-activity-and-sedentary-behaviour.
2. Lee IM, Shiroma EJ, Lobelo F, Puska P, Blair SN, Katzmarzyk PT. Effect of physical inactivity on major non-communicable diseases worldwide: an analysis of burden of disease and life expectancy. Lancet. 2012;380(9838):219-29.
3. Kohl HW, 3rd, Craig CL, Lambert EV, Inoue S, Alkandari JR, Leetongin G, et al. The pandemic of physical inactivity: global action for public health. Lancet. 2012;380(9838):294-305.
4. Guthold R, Stevens GA, Riley LM, Bull FC. Worldwide trends in insufficient physical activity from 2001 to 2016: a pooled analysis of 358 population-based surveys with 1·9 million participants. Lancet Glob Health. 2018;6(10):e1077-e86.
5. Al-Hazzaa HM. Physical inactivity in Saudi Arabia revisited: A systematic review of inactivity prevalence and perceived barriers to active living. Int J Health Sci (Qassim). 2018;12(6):50-64.
6. World Health Organization. Global action plan on physical activity 2018–2030: more active people for a healthier world. Geneva, World Health Organization; 2018.
7. Mandil AM, Alfurayh NA, Aljebreen MA, Aldukhi SA. Physical activity and major non-communicable diseases among physicians in Central Saudi Arabia. Saudi Med J. 2016;37(11):1243-50.
8. Salem V, AlHusseini N, Abdul Razack HI, Naoum A, Sims OT, Alqahtani SA. Prevalence, risk factors, and interventions for obesity in Saudi Arabia: A systematic review. Obes Rev. 2022;23(7):e13448.
9. Al-Quwaidhi AJ, Pearce MS, Sobngwi E, Critchley JA, O'Flaherty M. Comparison of type 2 diabetes prevalence estimates in Saudi Arabia from a validated Markov model against the International Diabetes Federation and other modelling studies. Diabetes Res Clin Pract. 2014;103(3):496-503.
10. Bauman AE, Reis RS, Sallis JF, Wells JC, Loos RJ, Martin BW. Correlates of physical activity: why are some people physically active and others not? Lancet. 2012;380(9838):258-71.
11. Rosselli M, Ermini E, Tosi B, Boddi M, Stefani L, Toncelli L, et al. Gender differences in barriers to physical activity among adolescents. Nutr Metab Cardiovasc Dis. 2020;30(9):1582-9.
12. Samara A, Nistrup A, Al-Rammah TY, Aro AR. Lack of facilities rather than sociocultural factors as the primary barrier to physical activity among female Saudi university students. International journal of women's health. 2015:279-86.
13. Al-Eisa ES, Al-Sobayel HI. Physical Activity and Health Beliefs among Saudi Women. J Nutr Metab. 2012;2012:642187.
14. Al-Otaibi HH. Measuring stages of change, perceived barriers and self efficacy for physical activity in Saudi Arabia. Asian Pac J Cancer Prev. 2013;14(2):1009-16.
15. Ammar A, Brach M, Trabelsi K, Chtourou H, Boukhris O, Masmoudi L, et al. Effects of COVID-19 Home Confinement on Eating Behaviour and Physical Activity: Results of the ECLB-COVID19 International Online Survey. Nutrients. 2020;12(6).
16. Barwais FA. Physical Activity at Home During the COVID-19 Pandemic in the Two Most-affected Cities in Saudi Arabia. The Open Public Health Journal. 2020;13:470-6.
17. Alfawaz H, Amer OE, Aljumah AA, Aldisi DA, Enani MA, Aljohani NJ, et al. Effects of home quarantine during COVID-19 lockdown on physical activity and dietary habits of adults in Saudi Arabia. Sci Rep. 2021;11(1):5904.
18. General Authority of Statistics. Saudi Census Riyadh, Saudi Arabia2022 [Available from: https://portal.saudicensus.sa/portal.
19. Faul F, Erdfelder E, Buchner A, Lang A-G. Statistical power analyses using G*Power 3.1: Tests for correlation and regression analyses. Behavior Research Methods. 2009;41(4):1149-60.
20. World Health Orgnization. Body mass index - BMI Copenhagen: WHO; 2015 [Available from: http://www.euro.who.int/en/health-topics/disease-prevention/nutrition/a-healthy-lifestyle/body-mass-index-bmi.
21. U.S. Department of Health and Human Services PHS, Centers for Disease Control and Prevention, National Center for Chronic Disease Prevention and Health Promotion, Division of Nutrition and Physical Activity,. Promoting Physical Activity: A Guide for Community Action. 1st ed. Champaign, IL: Human Kinetics; 1999.
22. Mayo Foundation for Medical Education and Research. Barriers to fitness Rochester, MIN: Mayo Foundation for Medical Education and Research; 2014 [updated 08/02/2014.
23. Team RC. R: A Language and Environment for Statistical Computing. 4.3.1 ed2023.
24. Burnham KP, Anderson DR. Multimodel inference: understanding AIC and BIC in model selection. Sociological methods & research. 2004;33(2):261-304.
25. Lau JH, Nair A, Abdin E, Kumarasan R, Wang P, Devi F, et al. Prevalence and patterns of physical activity, sedentary behaviour, and their association with health-related quality of life within a multi-ethnic Asian population. BMC Public Health. 2021;21:1-13.
26. O’Donoghue G, Perchoux C, Mensah K, Lakerveld J, van der Ploeg H, Bernaards C, et al. A systematic review of correlates of sedentary behaviour in adults aged 18–65 years: a socio-ecological approach. BMC Public Health. 2016;16(1):163.
27. Bauman AE, Reis RS, Sallis JF, Wells JC, Loos RJ, Martin BW. Correlates of physical activity: why are some people physically active and others not? The lancet. 2012;380(9838):258-71.

28. Al-Hazzaa HM. Physical inactivity in Saudi Arabia revisited: a systematic review of inactivity prevalence and perceived barriers to active living. International journal of health sciences. 2018;12(6):50.
29. Sofian M, Omar-Fauzee UPM, Abd-Latif R. The perspective of Arabic Muslim women toward sport participation. J Asia Pacific Studies. 2010;1:364-77.
30. Laar R, Zhang J, Yu T, Qi H, Ashraf MA. Constraints to women's participation in sports: A study of participation of Pakistani female students in physical activities. International journal of sport policy and politics. 2019;11(3):385-97.
31. Heard EM, Auvaa L, Conway BA. Culture X: addressing barriers to physical activity in Samoa. Health promotion international. 2017;32(4):734-42.
32. Cai Lian T, Bonn G, Si Han Y, Chin Choo Y, Chee Piau W. Physical activity and its correlates among adults in Malaysia: a cross-sectional descriptive study. PLoS One. 2016;11(6):e0157730.
33. Mathews E, Lakshmi J, Ravindran TS, Pratt M, Thankappan K. Perceptions of barriers and facilitators in physical activity participation among women in Thiruvananthapuram City, India. Global health promotion. 2016;23(4):27-36.
34. Baert V, Gorus E, Mets T, Geerts C, Bautmans I. Motivators and barriers for physical activity in the oldest old: a systematic review. Ageing research reviews. 2011;10(4):464-74.
35. Molanorouzi K, Khoo S, Morris T. Motives for adult participation in physical activity: type of activity, age, and gender. BMC public health. 2015;15:1-12.
36. Gaetano A. Relationship between physical inactivity and effects on individual health status. Journal of Physical Education and Sport. 2016;16(2):1069-74.
37. Karaca Çelik KE, Morales-Suárez-Varela M, Uçar N, Soriano JM, İnce Palamutoğlu M, Baş M, et al. Obesity prevalence, nutritional status, and physical activity levels in Turkish adults during the COVID-19 pandemic. Frontiers in Nutrition. 2024;11:1438054.
38. Li H, Zhang W, Yan J. Physical activity and sedentary behavior among school-going adolescents in low-and middle-income countries: insights from the global school-based health survey. PeerJ. 2024;12:e17097.
39. Gallardo LO, Esteban-Torres D, Rodríguez-Muñoz S, Moreno-Doña A, Abarca-Sos A. Is There Any Relationship between Physical Activity Levels and Academic Achievement? A Cross-Cultural Study among Spanish and Chilean Adolescents. Behavioral Sciences. 2023;13(3):238.
40. Shaw BA, Spokane LS. Examining the association between education level and physical activity changes during early old age. Journal of aging and health. 2008;20(7):767-87.
41. Hawkins MS, Storti KL, Richardson CR, King WC, Strath SJ, Holleman RG, et al. Objectively measured physical activity of USA adults by sex, age, and racial/ethnic groups: a cross-sectional study. International Journal of Behavioral Nutrition and Physical Activity. 2009;6(1):1-7.
42. Bu EQL, Chung HJ. Barriers towards exercise and physical activity of different ethnic groups in middle-aged female Singaporeans. Journal of exercise rehabilitation. 2018;14(5):739.
43. Arzu D, Tuzun EH, Eker L. Perceived barriers to physical activity in university students. Journal of sports science & medicine. 2006;5(4):615.
44. King AC, Kiernan M, Ahn DK, Wilcox S. The effects of marital transitions on changes in physical activity: results from a 10-year community study. Annals of Behavioral Medicine. 1998;20(2):64-9.

Click here to access/download
Supporting Information
PLOSOne_Human_Subjects_Research_Checklist.docx

Click here to access/download
Supporting Information
IRB-2021-018.pdf
