# Supplementary material for: Perceived barriers to physical activity and their predictors among adults in the Central Region in Saudi Arabia: Gender differences and cultural aspects
Source: PLoS One. 2025 Feb 7;20(2):e0318798. doi: 10.1371/journal.pone.0318798 (PMC11805373; doi:10.1371/journal.pone.0318798)
Supplement: Attachment — Submitted filename: Response to the Academic Editor.docx [file pone.0318798.s004.docx]

**Response to the Academic Editor**

Dear Professor Said,

Thank you for your thoughtful and constructive comments on our manuscript. We greatly appreciate your time and effort in reviewing our work. We have carefully considered all your points and revised the manuscript accordingly. Below, we provide a point-by-point response to your comments, detailing the changes made and providing justifications.

**1. Hypotheses Development**
**Editor’s Comment:** The hypotheses articulated in the manuscript are disconnected from the introduction and lack sufficient theoretical or empirical support.

**Response:** We agree that the link between the introduction and the hypotheses needs to be clearer. In the revised manuscript, we expanded the Introduction section to highlight the theoretical underpinnings and previous empirical findings that inform our hypotheses. Specifically, we have:

- Elaborated on how socio-ecological models and existing literature support the inclusion of factors such as education, income, and marital status as predictors of perceived barriers.
- Incorporated additional references (e.g., Bauman et al., 2012 [https://doi.org/10.1016/S0140-6736(12)60735-1]; Moreno-Llamas et al., 2022 [https://doi.org/10.1093/pubmed/fdab103]) that link sociodemographic and cultural factors to physical activity barriers.
- Revised the hypotheses to connect them to the cited literature explicitly. For example, we note that higher educational attainment has been associated with increased awareness and fewer barriers in some contexts. At the same time, in other settings, it correlates with more time constraints and sedentary occupations.

**2. Methods Section Structure**
**Editor’s Comment:** The Methods section should have a more transparent structure (Procedures, Participants, Data Collection Instruments, Statistics).

**Response:** We have reorganised the Methods section into four clear subsections:

- **Procedures:** We now present a step-by-step account of the study procedures, including the timeline, training of research assistants, and the approach used to contact potential participants at each entity.
- **Participants:** We have clarified inclusion and exclusion criteria and provided more details on the sampling method, the demographic attributes of participants, and how physical inactivity was determined.
- **Data Collection Instruments:** We have divided this into clear parts:
  - **Questionnaire Development and Validation:** Detailed the sources of the questionnaire items, the translation process, and the validation methods, including consultations with experts and the Content Validity Index.
  - **Reliability and Validity Assessments:** Provided Cronbach’s alpha and test-retest reliability results again clearly, ensuring all necessary details are in one place.
- **Statistical Analysis:** Clarified the statistical methods used, the rationale for logistic regression, and how we handled model selection, checking assumptions, and assessing model fit.

**3. Presentation and Use of Dashes**
**Editor’s Comment:** The manuscript contains multiple instances of points presented using dashes, which compromises clarity and coherence.

**Response:** We have revised all sections that previously relied on dash-listed points. These points have been converted into complete sentences or reorganised into paragraphs to improve clarity, flow, and adherence to formal academic style.

**4. Logistic Regression Metrics**
**Editor’s Comment:** Include additional model fit metrics and classification accuracy, such as Nagelkerke R² or pseudo-R².

**Response:** In the revised Methods section (Statistics), we now report the Nagelkerke R² values for the logistic regression models to estimate model fit. We also provide the classification discrimination power using the Area Under the Receiver Operating Curve. These additions help readers interpret the strength and utility of our models.

**5. Figures**
**Editor’s Comment:** Representing the same variable using frequency and percentage is unnecessary.

**Response:** We respectfully disagree with this comment as each graph shows a different aspect. The frequency graph shows the differences between males and females in the dataset. Therefore, we believe the comparison will be based on how each sex contributed to the dataset. The percentage graph adjusts the bar's heights to each sex's frequency and count. This plot acts as an adjustment to the imbalance between males and females. The final graph shows the total sample size with adjusting to sex. This provides an overall picture.

**6. Discussion Section Literature Engagement**
**Editor’s Comment:** The discussion section should engage more thoroughly with the existing literature, not just one or two references.

**Response:** We have enriched the Discussion section by referencing a broader range of studies, including those that offer divergent findings, to provide a more comprehensive understanding of our results in context. For example, we have included additional references on cultural barriers from similar settings. This expanded discussion highlights how our findings align with or contrast established literature, enhance credibility, and situate the results within the global research landscape. Moreover, we included more recent systematic reviews and meta-analyses that discussed the same topics, e.g., Corder et al., 2020 [ https://doi.org/10.1111/obr.12959].

**7. References**
**Editor’s Comment:** Some references require revision.

**Response:** We have rechecked all references for accuracy, formatting, and relevance. We have corrected any incomplete, outdated, or incorrectly formatted references. We have also ensured that all references now follow PLOS ONE style guidelines and that all URLs and DOIs are correctly included using EndNote 21.

**8. Language and Grammar**
**Editor’s Comment:** The manuscript should be reviewed by a native English speaker or professional editor.

**Response:** The revised manuscript was professionally copyedited to improve language fluency, clarity, and overall readability. The grammar, syntax, and style have been polished to meet academic standards.
